# Slick potassium channels limit TRPM3-mediated activation of sensory neurons

**DOI:** 10.3389/fphar.2024.1459735

**Published:** 2024-12-18

**Authors:** Patrick Engel, Fangyuan Zhou, Bang Tam Thi Tran, Achim Schmidtko, Ruirui Lu

**Affiliations:** Institute of Pharmacology and Clinical Pharmacy, Goethe University Frankfurt, Frankfurt, Germany

**Keywords:** heat nociception, TRPM3, Slick, sensory neurons, potassium current

## Abstract

Heat sensation is mediated by specialized heat-sensitive neurons in the somatosensory system that innervates the skin. Previous studies revealed that noxious heat sensation is controlled by the sodium (Na^+^)-activated potassium (K^+^) channel Slick (Kcnt2), which is highly expressed in nociceptive Aδ-fibers. However, the mechanism by which Slick modulates heat sensation is poorly understood. Here, we generated mice lacking Slick conditionally in sensory neurons expressing Nav1.8 (SNS-Slick^−/−^ mice). In SNS-Slick^−/−^ mice, the latency to express any nocifensive behavior was reduced in the hot plate and tail immersion tests. *In situ* hybridization experiments revealed Slick was highly co-expressed with the essential heat sensor, transient receptor potential (TRP) melastatin (TRPM) 3, but not with TRP vanilloid 1, TRP ankyrin 1, or TRPM2 in sensory neurons. Notably, SNS-Slick^−/−^ mice exhibited increased nocifensive behaviors following intraplantar injection of the TRPM3 activator pregnenolone sulfate. Patch-clamp recordings detected increased Na^+^-dependent outward K^+^ current (I_K_) after TRPM3 activation in sensory neurons, which showed no prominent I_K_ after the replacement of NaCl with choline chloride. Thus, our study suggests that Slick limits TRPM3-mediated activation of sensory neurons, thereby inhibiting noxious heat sensing.

## 1 Introduction

Potassium (K^+^) ion channels are a diverse group of ion channels that selectively enable the rapid diffusive flow of K^+^ across the plasma membrane, thereby shaping the neuron response. They play key roles in controlling neuronal activity and signal propagation throughout the nervous system (Ocana et al., 2004; Tsantoulas and McMahon, 2014). Slick (also known as K_Na_1.2 or Slo2.1; *Kcnt2* gene), a Na^+^-activated K^+^ (K_Na_) channel, is highly expressed in the nervous system and is involved in various physiological and pathological functions (Kaczmarek, 2013; Kaczmarek et al., 2017). Various studies, including ours, showed that mice lacking Slick globally (Slick^−/−^) exhibit increased nocifensive responses to noxious heat (Tomasello et al., 2017; Flauaus et al., 2022). However, the specific roles of Slick in noxious heat sensation remains unclear.

Noxious heat is detected by various heat-sensitive ion channels in the plasma membranes of sensory neurons with cell bodies located in the dorsal root ganglia (DRGs) and trigeminal ganglia. Several heat-sensitive members of the transient receptor potential (TRP) ion channels superfamily, including TRP vanilloid 1 (TRPV1), TRP ankyrin 1 (TRPA1), TRP melastatin (TRPM) 3 (TRPM3) and TRPM2, have been identified (Vandewauw et al., 2018; Vriens and Voets, 2018; Vilar et al., 2020). Deletion of TRPV1, TRPA1 and TRPM3 in mice alters the behavioral sensation of heat at noxious temperatures >45°C (Vandewauw et al., 2018). TRPM2 is activated at 30°C–40°C *in vivo* (Tan and McNaughton, 2016; Vilar et al., 2020). After these channels are activated by noxious heat, rapid influx of calcium (Ca^2+^) and sodium (Na^+^) causes depolarization of the membrane potential, leading to action potential firing in response to painful stimuli (Vangeel and Voets, 2019; Zhang et al., 2023). We hypothesized that Slick in sensory neurons can be functionally coupled to any of these essential heat sensors. To test this hypothesis, we generated tissue-specific knockout mice lacking Slick in sensory neurons and performed behavioral, electrophysiological, and tissue-staining experiments in this study. Our data suggest that Slick is functionally coupled to TRPM3 in sensory neurons.

## 2 Materials and methods

### 2.1 Animals

Slick^−/−^ and wild-type mice with C57BL/6 background were generated as previously described (Flauaus et al., 2022). To ablate Slick selectively in sensory neurons, floxed Slick (Slick^fl/fl^) mice (B6(129S4)-Kcnt2^tm1.1Clin^/J, JAX stock No. 028419; The Jackson Laboratory, United States) were crossed with SNS-Cre mice, which express the Nav1.8-driven Cre recombinase in nearly all primary nociceptive neurons (Agarwal et al., 2004), to obtain homozygous conditional Slick knockout mice (SNS-Slick^−/−^). Littermate Slick^fl/fl^ mice were used as controls. The mice were provided free access to food and water, and group-housed under a 12:12 light/dark cycle with controlled temperature (22°C ± 2°C) and humidity (55% ± 10%). Experiments were performed with 8- to 16-week-old animals. Littermate mice of both sexes were used in the behavioral studies. All experiments adhered to the Animal Research: Reporting on *In Vivo* Experiments guidelines and Replacement, Reduction, and Refinement principles, and were approved by our local Ethics Committee for Animal Research (Regierungspräsidium Darmstadt, Germany).

### 2.2 Real-time reverse transcription (RT)-polymerase chain reaction (PCR)

Mice were euthanized via carbon dioxide (CO_2_) inhalation, and lumbar (L4–L5) DRGs, lumbar (L4–L5) spinal cord, and prefrontal cortex were rapidly dissected, snap frozen in liquid nitrogen and stored at −80°C until use. Total RNA was isolated using the innuPREP Micro RNA Kit (#C-6134; Analytik Jena, Berlin, Germany) following the manufacturer’s instructions. Isolated RNA was quantified with a NanoDrop 2000 (Thermo Fisher Scientific, Dreieich, Germany), and cDNA was synthesized from 200 ng RNA using the first-strand cDNA synthesis kit (#10774691; Thermo Fisher Scientific) with random hexamer primers. qRT-PCR was performed with the CFX96 Touch Real-Time System (Bio-Rad, Hercules, Germany) using the iTaq Universal SYBR Green SuperMix (#1725120; Bio-Rad) and primer pairs (Biomers, Ulm, Germany) for Slick, TRPA1, TRPV1, TRPM3, and glyceraldehyde 3-phosphate dehydrogenase. All primer sequences used are listed in Table 1. Reactions were performed in duplicate by incubating for 2 min at 50°C and 10 min at 95°C, followed by 40 cycles of 15 s at 95°C and 60 s at 60°C. Water was used as a control to ensure specificity. Relative expression of the target gene levels was determined using the comparative 2^−ΔΔCT^ method and normalized to that of glyceraldehyde 3-phosphate dehydrogenase.

**TABLE 1:** List of primers, antibodies and *in situ* hybridization probes used.

List of RT-qPCR primers used	
Slick	fwd: 5′-gaa​agc​acc​atg​agt​gca​ga-3′; rev: 5′-gtt​ttg​aaa​gcg​cga​gag​ag-3′
Slack	fwd 5′-ctg​ctg​tgc​ctg​gtc​ttc​a-3′, rev 5′-aag​gag​gtc​agc​agg​ttc​aa-3′
TRPA1	fwd 5′-gga​aat​acc​cca​ctg​cat​tgt-3′, rev 5′-cag​cta​tgt​gaa​ggg​gtg​aca-3′
TRPV1	fwd 5′-act​ctt​acc​aca​cag​cag​cc-3′, rev 5′-gcc​caa​ttt​gca​acc​agc​ta-3′
TRPM3	fwd 5′-ggt​gtg​gct​tca​gga​gta​ct-3′, rev 5′-cca​gat​act​tgt​tca​cgc​cg-3′
TRPM2	fwd 5′-ctg​cgc​cta​gcg​atg​aga​tg-3′, rev 5′-cat​cct​gga​cat​act​ggt​ctg​c-3′
GAPDH	fwd 5′-caa​tgt​gtc​cgt​cgt​gga​tct-3′, rev 5′-gtc​ctc​agt​gta​gcc​caa​gat​g-3′

List of antibodies used	
mouse anti-neurofilament 200	NF200, 1:2000, #N0142, Sigma-Aldrich
rabbit anti-calcitonin gene-related peptide	CGRP; 1:800, #PC205L, Sigma-Aldrich
rabbit anti-vesicular glutamate transporter 3	VGLUT3, 1:100, #135203, Synaptic Systems
mouse anti-Slick	1:500, clone N11/33, #75-055, antibodiesinc
goat anti-mouse AF555	1:1000, #A21127. Thermo Fisher Scientific
goat anti-rabbit AF488	1:1000, #A11008. Thermo Fisher Scientific
Alexa Fluor 488-conjugated IB4	3.3 µg/ml, #121411, Thermo Fisher Scientific
Alexa Fluor 488-conjugated anti-tubulin β3	1:1000, #801203, BioLegend

List of *in situ* hybridization probes used (Thermo Fisher Scientific)	
type-1 probe for mouse Slick	1:40; #VB1-17744
type-1 probe for mouse scramble control	1:40; #VF1-17155
type-6 probe for mouse TRPA1	1:20; #VB6-18246
type-6 probe for mouse TRPV1	1:80; #VB6-16610
type-6 probe for mouse TRPM3	1:40; #VB6-3216456-VT
type-6 probe for mouse TRPM2	1:20; #VB6-3197141-01
type-6 probe for mouse scramble control	1:40; #VF6-18580

### 2.3 Immunohistochemistry and *in situ* hybridization

Mice were euthanized via CO_2_ inhalation and intracardially perfused with 0.9% saline, followed by 1% or 4% paraformaldehyde in phosphate-buffered saline (PBS) at pH 7.4. Lumbar (L4–L5) DRGs, sciatic nerves and lumbar (L4–L5) spinal cord were dissected, cryoprotected with 30% sucrose overnight, frozen in a tissue freezing medium (Tissue-Tek O.C.T. Compound; #4583; Sakura, Torrance, CA, United States) on dry ice, cut into 14- or 20-µm sections with a cryostat (CRYOSTAR NX50; Thermo Fisher Scientific) placed directly onto the adhesion microscope slide (#J1800AMNZ; Epredia, Breda, Netherlands), and stored at −80°C.

Immunohistochemistry was performed as previously described (Flauaus et al., 2022). The following primary antibodies were used: mouse anti-neurofilament 200 (NF200), rabbit anti-calcitonin gene-related peptide (CGRP), rabbit anti-vesicular glutamate transporter 3 (VGLUT3), and mouse anti-Slick. All antibodies used are listed in Table 1. After incubation with the primay antibodies, the sections were washed in PBS and incubated with the AlexaFluor-conjugated secondary antibodies (Thermo Fisher Scientific) diluted 1:1000. For *Griffonia simplicifolia* isolectin B4 (IB4) staining, the sections were incubated with Alexa Fluor 488-conjugated IB4 (3.3 μg/mL in PBS buffer containing 1 mM CaCl_2_⋅2H_2_O, 1 mM MgCl_2_, 1 mM MnCl_2_ and 0.2% Trion X-100, pH = 7.4) for 2 h at 4°C. For βIII-tubulin (TUBB3) staining, the sections were incubated with Alexa Fluor 488-conjugated anti-tubulin β3 antibody diluted in PBS for 2 h at 4°C. After immunostaining, the slides were washed with PBS, and cover-slipped with Fluoromount-G (No. 00–4958-02; Thermo Fisher Scientific). All antibodies used are listed in Table 1.

For *in situ* hybridization, the QuantiGene View-RNA tissue assay (Affymetrix, Thermo Fisher Scientific) was performed as previously described (Flauaus et al., 2022). Type-1 probe sets for mouse Slick, mouse scramble control, and type-6 probe sets for mouse TRPA1, TRPV1, TRPM3, TRPM2, and mouse scramble control were used. All probe sets used are listed in Table 1. In double *in situ* hybridization experiments, type-1 and type-6 labeled probes were simultaneously incubated. Finally, the sections were mounted using Fluoromount G.

Images were captured using the Eclipse Ni-U microscope (Nikon Europe B.V., Amsterdam, Netherlands) equipped with a monochrome DS-Qi2 camera (Nikon Europe B.V.). Specificity controls included omitting the first and/or the second primary antibodies, incubating type-1 and type-6 scramble probes, and incubating tissues of Slick^−/−^ mice.

For quantification of marker-positive neuron subpopulations in DRGs, serial sections of DRGs from SNS-Slick^−/−^ and control mice were cut, and 3–4 sections, at least 100 µm apart, per animal were counted. Only cells exhibiting clear staining above specificity control were included. The percentage of marker-positive DRG neurons is expressed as the ratio of marker-positive cells to number of pan-neuronal marker βIII-tubulin.

To quantify the intensity of Slick immunoreactivity in lamina I and outer lamina II of the dorsal horn, spinal cord sections of SNS-Slick^−/−^ and control mice were double-stained with antibodies to Slick and CGRP, and images were taken using the Eclipse Ni-U microscope. Grey value images were analyzed using ImageJ software (National institution Health, MD, United States). The area of CGRP-immunoreactivity was manually selected by the polygon selection tool and considered as the region of interest. The same region of interest was evaluated in the corresponding Slick image. With the analysis function, the mean gray value was measured in the defined region of interest.

### 2.4 Behavioral assays

All mice were acclimatized to the experimental environment for at least 2 days before the testing and were investigated by an observer blinded to the mouse genotype. All experiments were performed between 8:00 a.m. and 5:00 p.m.

#### 2.4.1 Rotarod test

The mice were tested for overall motor coordination and balance using a rotarod apparatus (Ugo Basile, Comerio, Italy) programmed gradually and uniformly to accelerate from 4 to 40 revolutions per minute for 300 s. The latency to fall off from the rotarod was recorded up to 300 s using a stopwatch. After the mice were trained for 3 consecutive days, three consecutive falls were recorded on the experiment day (Harada et al., 2017; Vandewauw et al., 2018).

#### 2.4.2 Hot plate test

Mice were individually confined to a Plexiglas cylinder on a heated metal surface (Hot/Cold Plate; Ugo Basile). The time interval between placement and observation of nocifensive behaviors (shaking or licking of the hindpaw, jumping) was recorded, and the mice were removed from the plate immediately after a notable response. To prevent tissue damage, temperatures of 47, 48, 49 and 50°C were applied with cut-off times of 90, 80, 70, and 60 s, respectively, as previously reported (Flauaus et al., 2022).

#### 2.4.3 Tail immersion test

Mice were immobilized in aluminum foil to allow free tail movement. For accommodation, the tip of the tail (approximately one-third of the tail length) was first immersed in a water bath (Sunlab D-8810; NeoLab, Germany) at 32°C for 20 s and then immersed in another water bath at 45, 47, 49, or 50°C, with cutoff times of 120, 60, 30, or 20 s, respectively. Then, the latency time to a tail withdrawal reflex was recorded. To prevent tissue damage, the tail was removed from the bath immediately after a notable response or upon reaching the cut-off time using a stopwatch (Vandewauw et al., 2018).

#### 2.4.4 Cold plate test

Mice were individually placed on a cold metal surface (Hot/Cold Plate; Ugo Basile) maintained at 10 or 5°C, and the total time the mouse spent lifting one or both forepaws over 60 s was recorded using a stopwatch (Luiz et al., 2019). Only one test per animal per day was performed at every indicated temperature.

#### 2.4.5 Von frey test

Mechanical thresholds were determined using von Frey filaments and the up-down method. The mice were individually placed in a chamber with a wire-mesh floor and acclimatized for 30 min before testing. Calibrated von Frey filaments (0.2–19.6 mN; 0.02–2.0 g; Ugo Basile) were applied to the plantar surface of the hindpaw, and measurements were started with the 0.6 g filament. Clear paw withdrawal, shaking, or licking during or immediately after the stimulus (up to 3 s after the filament was bowed) was defined as a nociceptive response. The 50% withdrawal thresholds were calculated using the online tool, “Up-down method for von Frey experiments” (https://bioapps.shinyapps.io/von_frey_app/) (Christensen et al., 2020).

#### 2.4.6 Pregnenolone sulfate (PS)-, capsaicin-, and allyl isothiocyanate (AITC)-induced nocifensive behavior

PS (5 nmol; #P162; Sigma-Aldrich, Darmstadt, Germany), capsaicin (5 μg; BML-EI125-0200; Enzo, Farmingdale, NY, United States), and AITC (10 mM; #36682; Sigma-Aldrich) in 20 µL 0.9% NaCl containing 0.5% dimethyl sulfoxide (DMSO) were injected into the plantar surface of the hind paw. After the injection, the behaviors of the mice were recorded using a video camera for 5 min in the absence of any observer. The video recordings were subsequently replayed, and the time spent licking, biting or lifting the injected paw was determined using a stopwatch by an observer blinded to the genotype.

### 2.5 Calcium imaging

Mice were euthanized with CO_2_, and lumbar (L1–L5) DRGs were quickly dissected and transferred to Hank’s Balanced Salt Solution without CaCl_2_ and MgCl_2_ (#14170088; Thermo Fisher Scientific) on ice. Primary cell cultures of lumbar DRG neurons for Ca^2+^ imaging were prepared as previously described (Zhou et al., 2022). Then, 20–26 h after primary cell culture preparation, the cells were loaded with 5 µM Fura-2-AM-ester (#50033; Biotium, Fremont, CA, United States) in Neurobasal A Medium (Thermo Fisher Scientific) for 45 min at 37°C. After loading, the coverslips were transferred to a perfusion chamber and continuously superfused with a physiological bath solution (145 mM NaCl, 5 mM KCl, 1.25 mM CaCl_2_, 1 mM MgCl_2_, 10 mM glucose, and 10 mM HEPES; adjusted to pH 7.4 with NaOH) at a flow rate of 1–2 mL/min. A Nikon Eclipse Ts2R inverse microscope equipped with a complete illumination system (DG4; Sutter Instruments, Novato, CA, United States), a Hamamatsu digital camera (ORCA-05G; Hamamatsu, Shizuoka Prefecture, Japan), Fura-2 filters, and a motorized microscope stage (Märzhäuser Wetzlar, Wetzlar, Germany) were used for calcium imaging. Images were taken every 2 s at two wavelengths (340 and 380 nm) and were processed using the NIS-Elements software (Nikon). Baseline measurements were performed in Ringer solution at a flow rate of 1–2 mL/min for 3 min.

For a functionality test of TRPM3, TRPA1, and TPPV1, the TRPM3 activator PS (100 µM), the TRPA1 agonist AITC (200 μM) and the TRPV1 activator capsaicin (100 nM) were dissolved in the bath solution from a 1,000 × stock solution in DMSO, and applied by bath perfusion for 20 s. At the end of each measurement, viable neurons were identified by the application of 75 mM KCl for 20 s. A Ca^2+^ response was defined as a simultaneous increase at 340 nm and a decrease at 380 nm, when the fluorescence ratio of 340 nm divided by 380 nm (F340/F380) normalized to baseline exceeded 20% of the baseline level. All experiments were performed at room temperature. Acquired images were displayed as the ratio of F340/F380.

### 2.6 Patch-clamp recording

Mice were euthanized with CO_2_, lumbar (L1–L5) DRGs were quickly dissected and transferred to dulbecco´s modified eagle´s medium containing 50 μg/mL gentamicin (Sigma-Aldllrich). After incubation with 500 U/mL collagenase IV and 2.5 U/mL dispase II for 30 min (both from Sigma-Aldrich), and 0.05% Trypsin/ethylenediaminetetraacetic acid (Thermo Fisher Scientific) for 10 min at 37°C, cells were washed twice with neurobasal medium supplemented with L-glutamine (2 mM) and 10% fetal calf serum. The cells were then mechanically dissociated using a pipette, seeded onto coverslips coated with poly-D-lysine-coated (200 μg/mL, Sigma-Aldrich) and cultured in TNB 100 medium supplemented with TNB 100 lipid protein complex, 100 μg/mL streptomycin, and penicillin (all from biochrom, Berlin, Germany) at 37°C and 5% CO_2_. To avoid neurite outgrowth, which could cause variations in expressed types and amounts of current, and to circumvent space clamp problems, the DRG neurons were studied within 28 h after plating.

To record the total outward K^+^ current (I_K_), the cultured coverslips were transferred to a recording chamber (RC-26G; Warner Instruments, Holliston, MA, United States) fitted to the stage of an up-right microscope (Axiovert 200; Zeiss, Oberkochen, Germany) and superfused with the extracellular solution. Physiological extracellular buffer contained 140 mM NaCl, 5 mM KCl, 2 mM CaCl_2_, 2 mM MgCl_2_ and 10 mM HEPES, adjusted to pH 7.4 with NaOH. For the Na^+^ free extracellular buffer, NaCl was replaced with 140 mM choline chloride, and adjusted to pH 7.4 with KOH. The pipette solution contained 140 mM KCl, 2 mM MgCl_2_, 5 mM EGTA, and 10 mM HEPES, adjusted to pH 7.4 with KOH (Lu et al., 2015; Lu et al., 2021). Recordings were conducted at room temperature with an EPC 9 patch-clamp amplifier combined with the Patchmaster software (HEKA Electronics, Lambrecht, Germany), and data were analyzed using the Fitmaster software (version 2 × 73.5, HEKA Electronics) and plotted using the GraphPad Prism software (version 10.0) for Windows (GraphPad, San Diego, CA, United States). The currents were filtered at 5 kHz and sampled at 20 kHz. The holding potential was −70 mV, and I_K_ was evoked by 500 ms voltage steps ranging from −120 to +120 mV in 20 mV increments. Patch micro-electrodes were fabricated with a Flaming/Brown micropipette puller (Sutter Instruments), and had a pipette resistance of 5–7 MΩ. Shortly before a coverslip was mounted for recordings, it was dipped in extracellular solution containing 3.3 μg/mL Alexa Fluor 488-conjugated Griffonia simplicifolia IB4 for 10 min, and only IB4-negative small-sized DRG neurons, larger than the IB4-positive DRG neurons were recorded. PS stock solution (100 μL, 500 µM in 20% DMSO) was added with a pipette to the bath chamber to reach a final concentration of 50 µM (Zhao and MacKinnon, 2023), and recordings were started 180 s thereafter. Afterwards, Na^+^ free extracellular buffer was infused into the bath chamber at a flow rate of 1–2 mL/min for 5 min and a recording was performed. All recordings were taken after stopping the superfusion system. PS-responsive neurons with reduced I_K_ after a 5-min washout with Na^+^ free extracellular buffer were used for analysis.

### 2.7 Statistical analysis

All statistical analyses were performed with the GraphPad Prism software (version 10.0; GraphPad Software). A probability value of *p* < 0.05 was considered statistically significant. No statistical power calculation or sample size calculation was performed before the study, however, the sample sizes employed are similar to those used in the field (Held et al., 2015; Lu et al., 2015; Flauaus et al., 2022; Zhou et al., 2022). The statistical test, statistical results, and group sizes are indicated in the figure legends or the main text. The results of the rotarod test are presented as median with interquartile range. All other data are shown as mean ± standard errors of the mean.

## 3 Results

### 3.1 Slick in sensory neurons inhibits the noxious heat sensing

**FIGURE 1:**
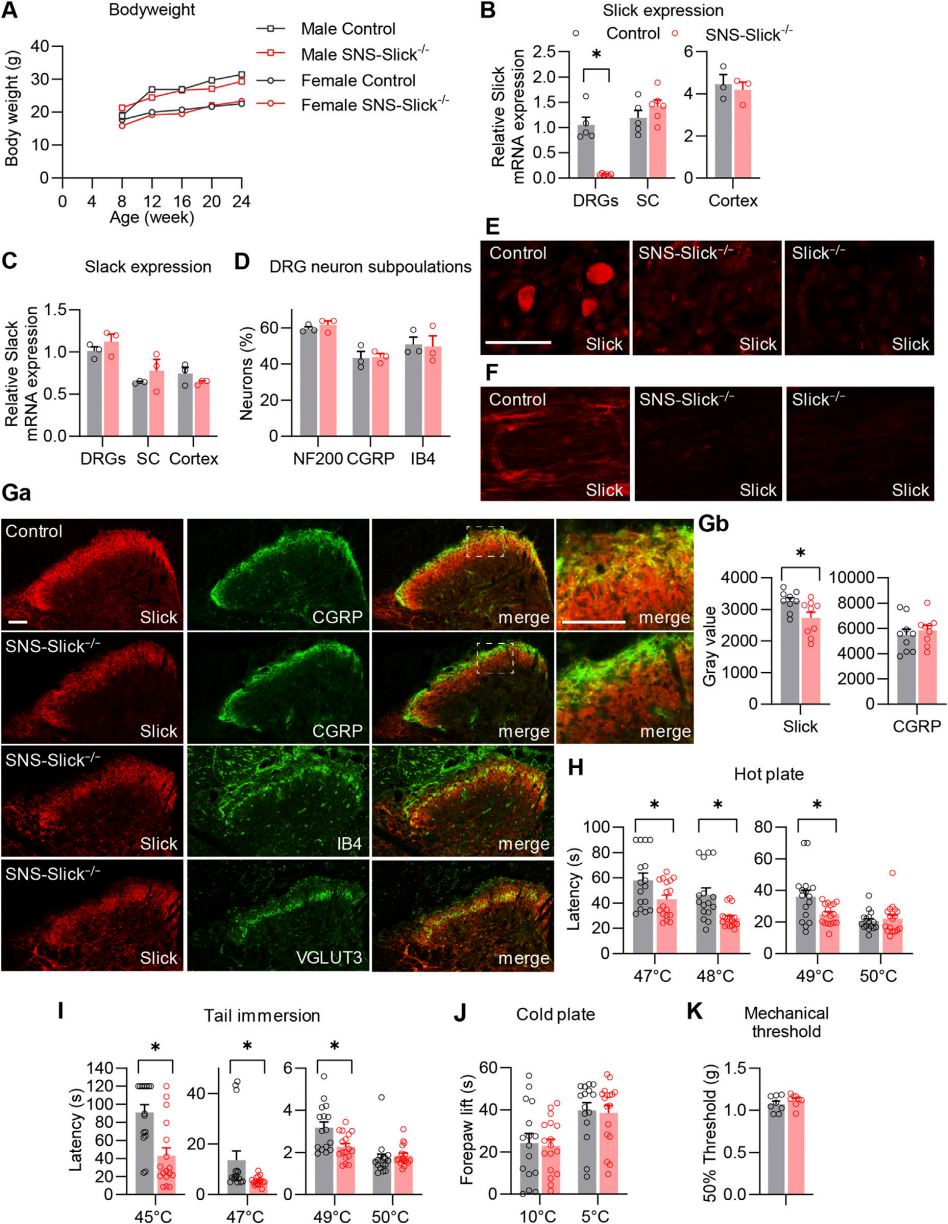
Tissue-specific deletion of Slick in sensory neurons affects heat sensation. **(A)** Body weight of SNS-Slick^−/−^ and control mice disaggregated by sex and age [SNS-Slick^−/−^: n = 14 (6 females and 8 males); control: n = 13 (6 females and 7 males)]. **(B)** Quantitative reverse transcription-polymerase chain reaction (qRT-PCR) analyses revealed that Slick mRNA levels were considerably reduced in the dorsal root ganglia [DRGs; *p* = 0.008; SNS-Slick^−/−^: n = 6 (3 females and 3 males); control: n = 5 (3 females and 2 males)], but unaltered in the spinal cord [SC; *p* = 0.454; SNS-Slick^−/−^: n = 6 (3 females and 3 males); control: n = 5 (3 females and 2 males)] and prefrontal cortex [*p* = 0.670; SNS-Slick^−/−^: n = 3 (2 females and 1 male); control: n = 3 (2 females and 1 male)] of SNS-Slick^−/−^ mice. **(C)** Slack mRNA levels in DRGs (*p* = 0.352), SC (*p* = 0.401) and prefrontal cortex (*p* = 0.299) are similar in SNS-Slick^−/−^ and littermate control mice [SNS-Slick^−/−^: n = 3 (2 females and 1 male); control: n = 3 (2 females and 1 male)]. **(D)** Percentages of sensory neurons immunoreactive to neurofilament 200 (NF200; *p* = 0.451) or calcitonin gene-related peptide (CGRP; *p* = 0.903), and binding isolectin B4 (IB4; *p* = 0.873), were similar in SNS-Slick^−/−^ mice [n = 3 (2 females and 1 male)] and littermate controls (n = 4 females). **(E, F)** Slick immunostaining of DRGs **(E)** and sciatic nerves **(F)** of control, SNS-Slick^−/−^, and Slick^−/−^ mice revealed specific Slick immunoreactivity in the tissues of control mice. **(G) (Ga)** Double-staining of Slick and markers [CGRP, IB4, and vesicular glutamate transporter 3 (VGLUT3)] in the spinal dorsal horn of control and SNS-Slick^−/−^ mice. Areas marked by white boxes are shown at higher magnification on the right. **(Gb)** Mean grey values of Slick and CGRP immunofluorescence in lamina I and outer lamina II of the spinal dorsal horn [*p* = 0.023; n = 9 section per group from 5 (4 females and 1 male) SNS-Slick^−/−^ mice and 8 (3 females and 5 males) control mice]. These data revealed that Slick immunoreactivity in lamina I and outer lamina II (marked by CGRP staining) was reduced in SNS-Slick^−/−^ mice compared to that in control mice. **(H)** Hot plate tests revealed significantly reduced latencies in SNS-Slick^−/−^ mice at 47°C (*p* = 0.043), 48°C (*p* = 0.002), and 49°C (*p* = 0.023) but not at 50°C [*p* = 0.950; SNS-Slick^−/−^: n = 17 (7 females and 10 males); control: n = 16 (8 females and 8 males)]. **(I)** Tail immersion tests showed significantly reduced latencies in SNS-Slick^−/−^ mice at 45°C (*p* < 0.001), 47°C (*p* = 0.006), and 49°C (*p* = 0.007) but not at 50°C [*p* = 0.176; SNS-Slick^−/−^: n = 17 (6 females and 11 males); control: n = 16 (7 females and 9 males)]. **(J)** In cold plate tests, SNS-Slick^−/−^ and control mice exhibited similar forepaw lift times at temperatures of 10°C (*p* = 0.812) and 5°C [*p* = 0.809; SNS-Slick^−/−^: n = 17 (6 females and 11 males); control: n = 16 (7 females and 9 males)]. Unpaired *t*-test. **(K)** In the von Frey test, SNS-Slick^−/−^ and control mice exhibited normal mechanical thresholds [*p* = 0.231; SNS-Slick^−/−^: n = 8 (5 females and 3 males); control: n = 11 (6 females and 5 males)]. Unpaired *t*-test. Scale bars: 50 µm **(E, F)** and 25 µm **(G)**. *p < 0.05. In **(A)** two-way repeated-measures analysis of variance with Sidak’s multiple comparison test was used. In **(B–D, H–J)**, multiple unpaired *t*-test was used. In **(K)**, unpaired t-test was used. All data are presented as means ± standard errors of the mean.

To investigate the functional role of Slick in the peripheral nervous system in pain processing *in vivo*, we generated mice that specifically lack Slick in nociceptive sensory neurons. For this purpose, we crossed mice carrying the floxed *Kcnt2* gene (Martinez-Espinosa et al., 2015) with SNS-Cre mice expressing Cre under the control of Scn10a (Na_V_1.8) promoter (Agarwal et al., 2004). The resulting conditional knockout mice (SNS-Slick^−/−^ mice) were viable and fertile, with no gross physical or behavioral defects compared to the littermate controls. No difference in body weight was observed between SNS-Slick^−/−^ and control mice (Figure 1A). To confirm the selective ablation of Slick in the DRGs of SNS-Slick^−/−^ mice, we analyzed Slick expression levels in lumbar DRGs, lumbar spinal cord, and prefrontal cortex via qRT-PCR. Indeed, *Slick* mRNA levels were significantly reduced in the DRGs of SNS-Slick^−/−^ mice compared to those in the DRGs of control mice (Figure 1B), but similar in the spinal cord and prefrontal cortex (Figure 1B) of both genotypes, confirming the specific deletion of *Slick* in sensory neurons. The expression of Slack (Kcnt1), which encodes the K_Na_1.1 subunits that are homologous to Slick, was not compensatorily regulated in the lumbar DRGs, lumbar spinal cord, and prefrontal cortex of SNS-Slick^−/−^ mice (Figure 1C). Furthermore, *Slick* deletion in sensory neurons did not affect the general structural properties of sensory neurons as the overall frequencies of DRG neuron populations positive for standard markers (NF200, CGRP, and IB4) were similar between the genotypes (Figure 1D).

Immunostaining using a specific anti-Slick antibody showed clear Slick immunoreactivity in the DRG neurons and the sciatic nerves of control mice, but not of SNS-Slick^−/−^ and Slick^−/−^ mice (Figures 1E, F). These data not only confirmed the specific deletion of Slick in the sensory neurons of SNS-Slick^−/−^ mice (Figure 1E), but also indicate that Slick protein is present in the sciatic nerve (Figure 1F). We also detected Slick protein in the spinal cord of SNS-Slick^−/−^ and control mice (Figure 1Ga). With the deletion of *Slick* in the central terminals of sensory neurons in the superficial dorsal horn, Slick immunoreactivity was significantly reduced in lamina I and outer lamina II of the spinal dorsal horn (indicated by CGRP staining; Figure 1Gb) but not in the dorsal region of inner lamina II (indicated by IB4 staining), and ventral region of inner lamina II (indicated by vesicular glutamate transporter-3 staining). Slick immunoreactivity observed in the spinal cord of SNS-Slick^−/−^ mice (Figure 1G) most likely represents dorsal horn neurons that express Slick (Flauaus et al., 2022). Together, these data indicate that *Slick* is specifically deleted in sensory neurons of SNS-Slick^−/−^ mice.

As a prerequisite for behavioral testing, we investigated the motor function of SNS-Slick^−/−^ mice and control mice using an accelerating rotarod test. SNS-Slick^−/−^ mice demonstrated intact motor coordination and balance as compared to the control mice (median fall-off latencies: SNS-Slick^−/−^, 300 s [interquartile range: 272.0–300.0 s]; control, 296 s [interquartile range: 254.0–300.0 s]; *p* = 0.253; Mann-Whitney U test; n = 16–17 mice/group), indicating that SNS-Slick^−/−^ mice are suitable for behavioral profiling of animal pain models. Next, we tested heat sensation in SNS-Slick^−/−^ and control mice using the hot plate test. Compared to the littermate control mice, SNS-Slick^−/−^ mice demonstrated significantly shorter latency times to noxious heat on the plate at 47, 48, and 49°C, but a normal latency time at 50°C (Figure 1H). Similarly, in the tail immersion test, SNS-Slick^−/−^ mice demonstrated shorter latency times at 45, 47, and 49°C (Figure 1I), but normal latency time at 50°C (Figure 1I). In contrast, SNS-Slick^−/−^ mice showed normal responses in the cold plate test at 10°C and 5°C (Figure 1J) and normal mechanical thresholds (Figure 1K). We analyzed female and male mice in the behavior tests including hot plate and tail immersion tests; the data disaggregated by sex are presented in Supplementary Figures S1A, B. However, we did not analyze the effect of sex as we were not powered to detect sex differences. Our finding suggest that specific ablation of Slick in sensory neurons enhances responses to noxious heat.

### 3.2 Slick in sensory neurons inhibits TRPM3 activation-induced nocifensive behavior

**FIGURE 2:**
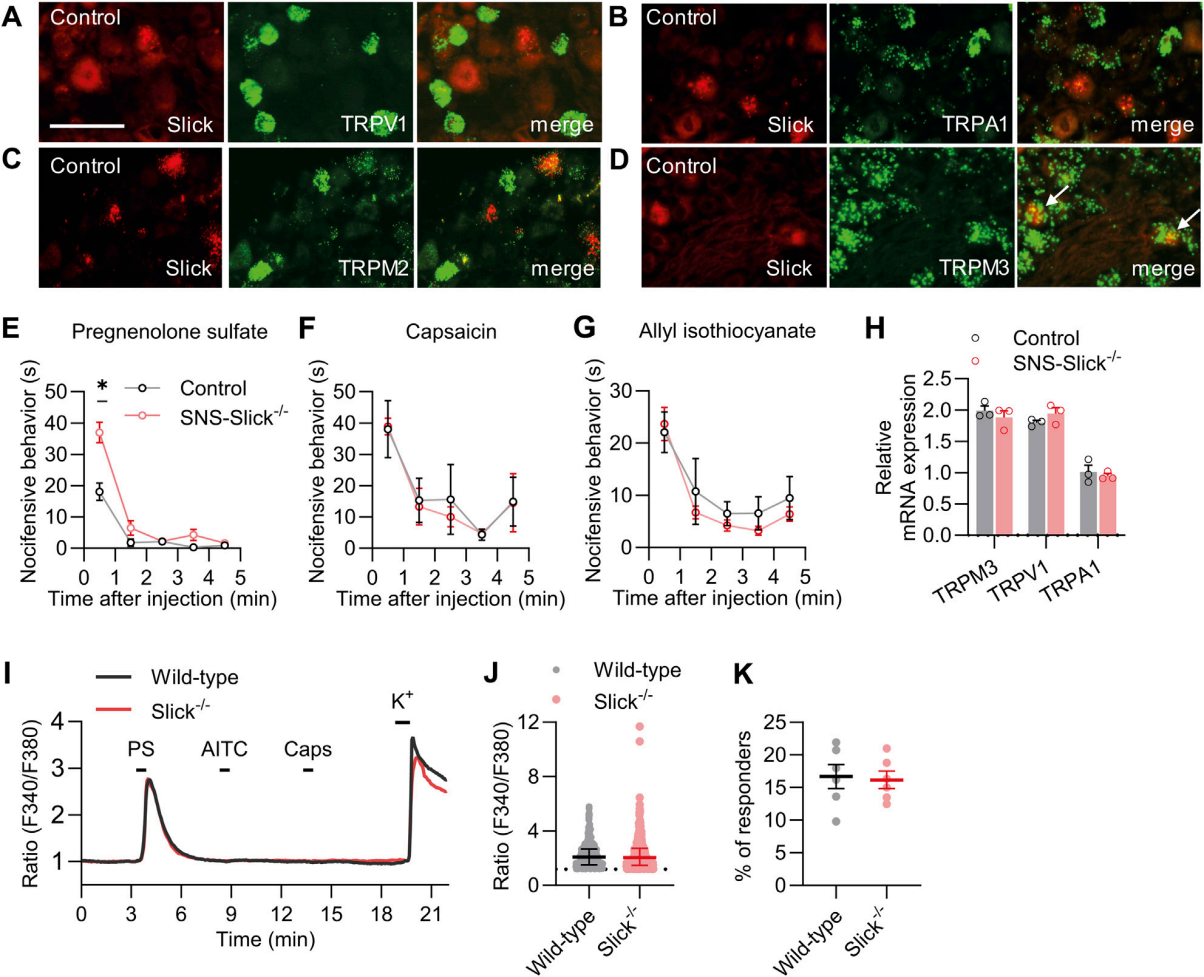
Slick is involved in transient receptor potential melastatin 3 (TRPM3) activation-induced nocifensive behavior. **(A–D)** Double *in situ* hybridization of *Slick* and *TRPV1* **(A)**, *TRPA1* **(B)**, *TRPM2* **(C)**, and *TRPM3* **(D)** mRNAs in the DRGs of control mice. Colocalization of Slick and TRPM3 is indicated by the white arrows. **(E)** SNS-Slick^−/−^ mice showed enhanced nocifensive behaviors in the first minute after the intraplantar injection of pregnenolone sulfate [PS; *p* = 0.004; SNS-Slick^−/−^: n = 8 (4 females and 4 males); control: n = 8 (4 females and 4 males)]. **(F, G)** Nocifensive behaviors of SNS-Slick^−/−^ mice were unaltered after the intraplantar injection of capsaicin (F; SNS-Slick^−/−^: n = 5 [2 females and 3 males]; control: n = 5 [2 females and 3 males]) or allyl isothiocyanate (G; AITC; SNS-Slick^−/−^: n = 8 [5 females and 3 males]; control: n = 8 [5 females and 3 males]). **(H)** qRT-PCR analyses revealed that *TRPM3* (*p* = 0.458), *TRPV1* (*p* = 0.250), and *TRPA1* mRNA (*p* = 0.610) levels are similar in the DRGs of SNS-Slick^−/−^ (n = 3 [2 females and 1 male]) and littermate control (n = 3 [2 females and 1 male]) mice. **(I)** Representative examples of Fura-2-ratiometric calcium traces in sensory neurons of wild-type and Slick^−/−^ mice that reacted to a stimulation with PS and KCl, but not with capsaicin or AITC. **(J)** Ratio of the calcium response to PS stimulation in sensory neurons from wild-type (n = 495 neurons in 6 mice [3 females and 3 males]) and Slick^−/−^ mice (n = 460 neurons in 6 mice [3 females and 3 males]). *p* = 0.505. **(K)** Percentage of sensory neurons from wild-type and Slick^−/−^ mice that responded to the PS stimulation, but not to the AITC or capsaicin stimulation (n = 6 mice [Slick^−/−^: 3 females and 3 males; WT: 3 females and 3 males]). *p* = 0.821. Scale bar: 50 μm *p < 0.05. In **(E–G)**, two-way repeated-measures analysis of variance with Sidak’s multiple comparison test was used. In **(H)**, multiple unpaired *t*-test was used. In **(J, K)**, unpaired t-test was used. Data are presented as means ± standard errors of the mean.

Next, we explored whether the increased heat sensitivity of SNS-Slick^−/−^ mice is related to the functional coupling with essential heat detectors in sensory neurons. Acute noxious heat sensing in mice depends on a triad of TRP ion channels: TRPV1, TRPA1, and TRPM3 (Vandewauw et al., 2018). In addition to these channels, TRPM2 is a thermally activated ion channel in the peripheral sensory neurons (Tan and McNaughton, 2016; Vilar et al., 2020). To estimate the possible interaction of Slick with these TRP channels, we performed double *in situ* hybridization experiments using lumbar DRG sections. These experiments showed that Slick did not colocalize with TRPV1, TRPA1, and TRPM2 in sensory neurons (Figures 2A–C). However, Slick was expressed in a subset of TRPM3-expressing sensory neurons (89.1% ± 4.4% of Slick-positive neurons expressed TRPM3, whereas 35.0% ± 6.2% of TRPM3-positive neurons expressed Slick; Figure 2D). These data support our hypothesis that Slick might be functionally coupled to TRPM3 in sensory neurons.

In addition to its role in heat sensation, TRPM3 acts as a nociceptor sensitive to various physical and chemical stimuli, most notably the endogenous neurosteroid PS (Wagner et al., 2008; Bamps et al., 2021). To explore whether TRPM3 is functionally coupled to Slick in sensory neurons, we tested the nocifensive behavior of SNS-Slick^−/−^ and control mice following the intraplantar injection of PS (Vriens et al., 2011). Interestingly, nocifensive behavior induced by PS injection was significantly enhanced in SNS-Slick^−/−^ mice compared to that in control mice in the first minute after injection, suggesting that Slick limits the nocifensive behavior induced by activation of TRPM3 in sensory neurons (Figure 2E). We analyzed female and male mice in the PS test and present the data disaggregated by sex in Supplementary Figure S1C. As control experiments, we intraplantarly injected TRPV1 activator capsaicin and TRPA1 activator AITC in SNS-Slick^−/−^ and control mice. Unlike the TRPM3-mediated nocifensive behavior, the nocifensive responses to capsaicin and AITC were unaltered in SNS-Slick^−/−^ mice (Figures 2F, G). Further control experiments using qRT-PCR revealed that the mRNA levels of *TRPM3*, *TRPV1*, and *TRPA1* were similar in DRGs of SNS-Slick^−/−^ and control mice (Figure 2H). These results indicate that Slick in sensory neurons is functionally coupled to TRPM3 but not TRPV1 and TRPA1.

We then investigated whether the enhanced PS-induced nocifensive behavior in SNS-Slick^−/−^ mice is related to altered TRPM3-dependent Ca^2+^ influx into sensory neurons. We compared the PS-induced changes in intracellular Ca^2+^ in cultured DRG neurons of wild-type and Slick^−/−^ mice via Ca^2+^ imaging. As TRPM3, TRPV1, and TRPA1 exhibit largely overlapping expression profiles (Vriens et al., 2011; Vandewauw et al., 2018) and Slick is only expressed in a subpopulation of TRPM3-positive sensory neurons (Figure 2D) but not in the TRPV1- or TRPA1-expressing sensory neurons (Figures 2A,B), we consecutively stimulated the cultured DRG neurons with 100 μM PS, 200 µM AITC, and 100 nM capsaicin (each for 20 s) and analyzed only those neurons that reacted to PS, but not to AITC and capsaicin (Figure 2I). Notably, average value to peak amplitudes induced by PS stimulation (Figure 2J) and percentage of neurons only responsive to PS (Figure 2K) were indistinguishable between the wild-type and Slick^−/−^ mice. Therefore, TRPM3-dependent Ca^2+^ transients were not modulated by Slick in sensory neurons.

### 3.3 TRPM3 activation alters Slick-mediated potassium currents in sensory neurons

**FIGURE 3:**
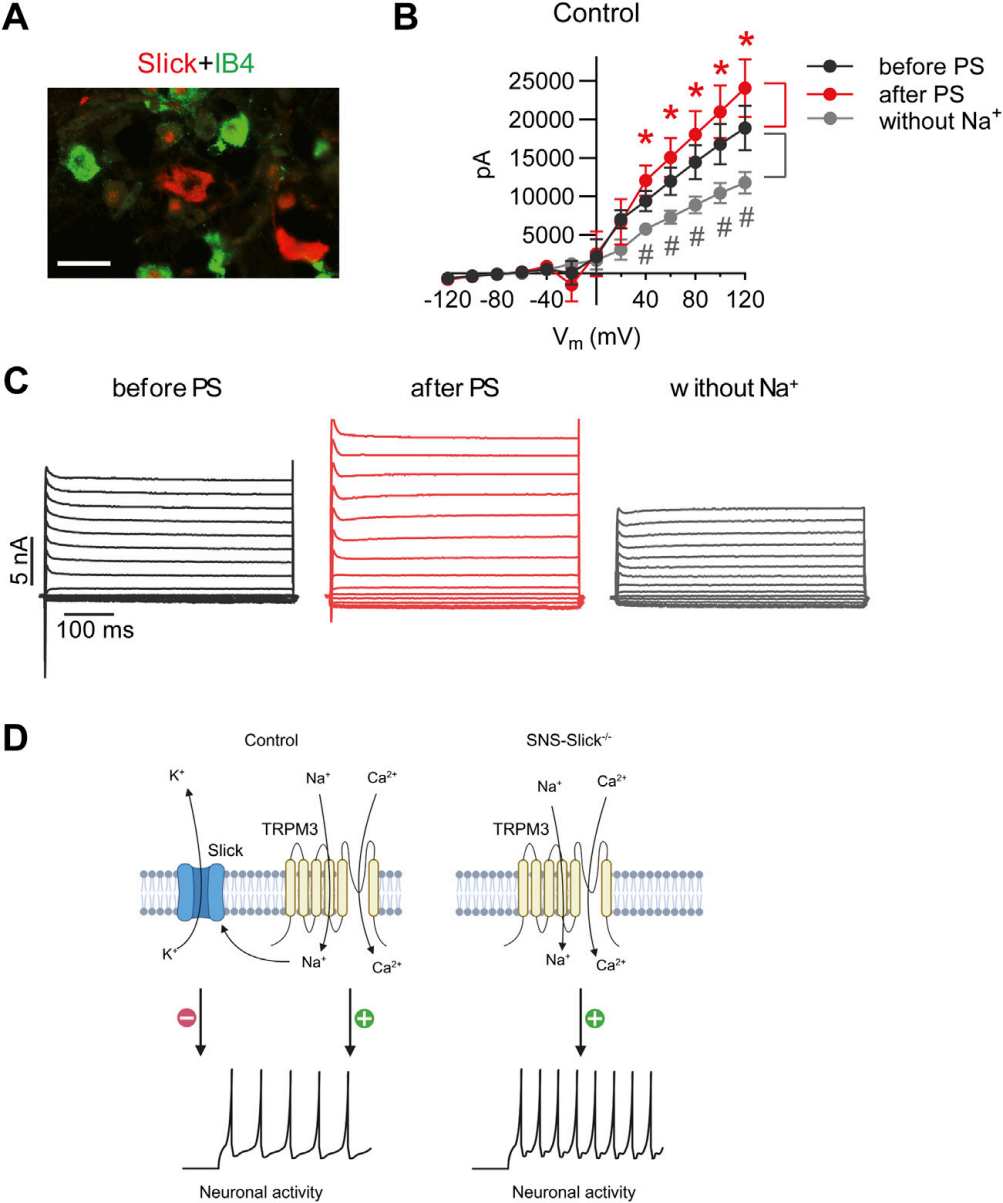
PS-mediated modulation of potassium currents in the isolectin B4 (IB4)-negative sensory neurons of control mice. **(A)** Double immunostaining of Slick and IB4 in the lumbar DRGs of wild-type mice revealed that Slick is expressed in IB4-negative small-diameter DRG neurons. **(B)** IV relations of outward potassium currents (I_K_) determined in whole-cell patch-clamp recordings on DRG neurons of twelve wild-type mice at baseline, after PS (50 µM) application in the physiological extracellular buffer, and after 5 min washout using an extracellular buffer without Na^+^. In total, 56 DRG neurons from twelve mice were analyzed. Shown are the recordings of the cells (n = 7) that exhibited increased I_K_ after PS application (indicating that the cell was TRPM3-positive) and decreased I_K_ in the Na^+^-free buffer (indicating that the cell was Slick-positive). *p < 0.05 indicates significantly increased I_K_ after PS application (after PS). ^#^ *p* < 0.05 indicates significantly decreased I_K_ after Na^+^ replacement (without Na^+^) compared to that before PS application (before PS). **(C)** Representative I_K_ traces before and after PS application in the physiological extracellular buffer and after washout with the Na^+^-free buffer. **(D)** Schematic diagram demonstrating the functional coupling of Slick and TRPM3 in IB4-negative small-diameter sensory neurons. TRPM3 activation by heat or PS leads to an influx of Na^+^ and Ca^2+^ into sensory neurons to activate neuronal activity. Increased Na^+^ influx activates Slick, which results in K^+^ efflux and thus inhibits neuronal activity. **(D)** is created with BioRender.com/z68j113. Scale bar: 100 µm. In **(B)**, multiple paired *t*-test was used. Data are presented as means ± standard errors of the mean.

Next, we performed patch-clamp recordings of sensory neurons to explore the interaction between Slick and TRPM3. We previously reported that the cellular distribution of Slick in DRGs is restricted to neurons not binding to IB4 but positive for both NF200 and CGRP (Flauaus et al., 2022). As shown in Figure 3A, Slick-positive DRG neurons did not overlap with the IB4-positive neurons. Therefore, we performed whole-cell patch-clamp recordings of IB4-negative small-diameter DRG neurons of control mice to analyze the I_K_ currents. For each neuron, three consecutive recordings were performed: (1) at the baseline, (2) after adding PS to the bath chamber, and (3) after 5-min washout and replacement of NaCl with choline chloride (to obtain an Na^+^-free solution) in the extracellular buffer in order to detect I_K_ currents driven by the Na^+^-activated K^+^ channel. To identify the Slick-positive cells, we calculated the ratio of I_K_ current at the baseline (before PS) and after the replacement of NaCl with choline chloride (without Na^+^) at +80 mV. Neurons with a prominent reduction in I_K_ current by more than 20% after the removal of extracellular Na^+^ were defined as Slick-positive cells. We patched 56 sensory neurons from twelve control mice and identified seven neurons responding to PS and with reduced I_K_ after Na^+^ replacement. In these seven neurons, a prominent reduction in I_K_ peak amplitude with a linear I–V relationship at positive potentials ranging from +40 to +120 mV was detected (Figure 3B), suggesting the presence of a large Na^+^-activated K^+^ current. Notably, the amplitude of I_K_ significantly increased after the addition of PS, with a linear I–V relationship at positive potentials ranging from +40 to +120 mV as compared to the amplitude of I_K_ before PS (Figure 3B). Representative examples of I_K_ currents evoked by PS and NaCl replacement are shown in Figure 3C. Together, these data suggest that TRPM3 activation by PS in sensory neurons leads to Na^+^ influx, thereby activating Slick to limit further neuronal activation (Figure 3D).

## 4 Discussion

This study demonstrated that Slick in sensory neurons plays a functional role in heat sensation. Our *in situ* hybridization experiments revealed that most of Slick-positive sensory neurons expressed the essential heat sensor, TRPM3. At the behavioral level, SNS-Slick^−/−^ mice showed reduced latency time to express any nocifensive behavior in the hot plate and tail immersion tests, and greater nocifensive responses after the intraplantar injection of a TRPM3 activator. At the cellular level, activation of TRPM3 increased the Na^+^-dependent I_K_ currents in sensory neurons, which are driven by Slick in TRPM3-positive cells. These findings suggest that Slick exerts specific inhibitory effects upon TRPM3 activation in sensory neurons. The main findings from this study are highlighted in a schematic diagram in Figure 3D.

TRPM3 is highly expressed in a proportion of small-diameter DRG neurons, which have a size distribution similar to that of capsaicin-sensitive neurons (Vriens et al., 2011). Despite the limited expression of Slick in DRG neurons (only approximately 9% of total DRG neurons express Slick) and its enrichment in CGRP; NF200-positive Aδ-fiber nociceptors (Flauaus et al., 2022), Slick was almost exclusively expressed in the TRPM3-positive neurons, but not in the TRPV1-, TRPA1-, and TRPM2-positive neurons. TRPM3 expressed in sensory neurons facilitates noxious heat sensation and plays key roles in inflammatory hyperalgesia and neuropathic pain (Thiel et al., 2017; Behrendt, 2019; Aloi et al., 2023). Therefore, TRPM3 is a promising novel target for pain treatment. Using another Slick^−/−^ mouse line, Tomasello et al. reported that Slick^−/−^ mice exhibited increased thermal hyperalgesia during chronic inflammatory pain and neuropathic pain (Tomasello et al., 2017). However, Slick is highly expressed in spinal dorsal horn neurons (Flauaus et al., 2022), possibly contributing to chronic pain. Therefore, further studies are necessary to elucidate the functional roles of Slick in sensory and dorsal horn neurons in chronic pain.

Heterologous expression of the Slick channel indicates that it is predominantly activated by intracellular Na^+^ and Cl^−^ (Bhattacharjee et al., 2003). TRPM3 is a non-selective cation channel that is permeable for Ca^2+^, Mg^2+^, and Zn^2+^ via the central pore (Oberwinkler et al., 2005; Wagner et al., 2010). In addition to the central pore, an alternative ion permeation pathway has been identified in TRPM3. This alternative pathway allows massive Na^+^ influx at negative voltages, thereby enhancing neuronal excitation and exacerbating TRPM3-dependent pain (Vriens et al., 2014; Vriens and Voets, 2018). Because Slick is a K_Na_ channel, the opening of Slick can lead to action potential repolarization in an intracellular Na^+^ dependent manner (Gao et al., 2008). Despite the well-established existence of the Slick channel, the circumstances under which cytosolic Na^+^ elevation arising from physiological stimuli is sufficient to produce Slick activation remain unclear. Therefore, we can only speculate that TRPM3 activation-induced Na^+^ influx may be responsible for Slick activation and subsequent inhibition of the processing of noxious thermal stimuli <50°C. In our patch-clamp experiments, TRPM3 was stimulated by PS applied to the extracellular buffer, because PS is membrane-impermeant and the PS-interacting domain is located at the extracellular side (Wagner et al., 2008; Held et al., 2015). Notably, TRPM3 has an unusually large number of splice variants (Oberwinkler et al., 2005), and PS activates TRPM3α2 to produce both outward and inward current (Vriens et al., 2011; Held et al., 2015; Van Hoeymissen et al., 2020; Hossain Saad et al., 2021). Unfortunately, our experimental setup does not allow us to distinguish between PS-induced outward and inward currents. Therefore, the increased I_K_ current after PS possibly includes the TRPM3 currents. However, the proportions of Slick- and TRPM3-mediated currents remain unclear, warranting further investigation.

The ion channel modulation by noxious heat has been addressed in several reviews (Palkar et al., 2015; Vriens and Voets, 2018; Lamas et al., 2019; Kashio and Tominaga, 2022). In addition to the channels tested in this study, Ca^2+^-activated Cl^−^ channel anoctamin 1 (ANO1) also acts as a heat sensor in DRG neurons (Cho et al., 2012; Lee et al., 2014). ANO1 activation in DRG neurons causes depolarization, thus firing the action potential (Liu et al., 2010; Cho et al., 2012; Lee et al., 2014). Mice lacking ANO1 in DRG neurons exhibit significantly increased withdrawal latencies in the tail immersion test at 50°C–54°C (Cho et al., 2012). Notably, ANO1 is barely expressed in peptidergic nociceptors (Usoskin et al., 2015). Here, SNS-Slick^−/−^ mice exhibited short latencies in the tail immersion test at temperatures <50°C; therefore, an interaction between Slick and ANO1 seems unlikely. Recently, a novel sensor of noxious heat, the transmembrane channel-like (TMC) 6 has been identified. Mice selectively lacking TMC6 in the sensory neurons exhibited longer latencies to noxious heat on the hot plate at 48°C–56°C. However, TMC6-mediated noxious heat-elicited Ca^2+^ response and action potential firing, but did not affect Na^+^ current (Zhang et al., 2024). Therefore, it is less likely that Slick modulates heat sensation via TMC6. These findings suggest that Slick specifically limits TRPM3-mediated activation of sensory neurons upon noxious heat stimulation.

In conclusion, our observations indicate that Slick in sensory neurons exerts a critical inhibitory function in the processing of noxious heat sensing.

## Data Availability

The raw data supporting the conclusions of this article will be made available by the authors, without undue reservation.

## Supplementary material

SUPPLEMENTARY FIGURE S1 Data of key behavior experiments disaggregated by sex. Data correspond to those presented in Figures 1H, 1I, and 2E, but are separated in male and female cohorts. Behavioral responses of SNS-Slick^−/−^ mice in the hot plate test **(A)**, in the tail immersion test **(B)** and pregnenolon sulfate **(C)**. **P* < 0.05. In Figure 1A, B multiple unpaired *t*-test was used. In Figure 1C two-way repeated-measures analysis of variance with Sidak’s multiple comparison test was used. Data are presented as means ± standard errors of the mean.
