# Supplementary figures and images for: Slick potassium channels limit TRPM3-mediated activation of sensory neurons
Source: Front Pharmacol. 2024 Dec 18;15:1459735. doi: 10.3389/fphar.2024.1459735 (PMC11688182; doi:10.3389/fphar.2024.1459735)

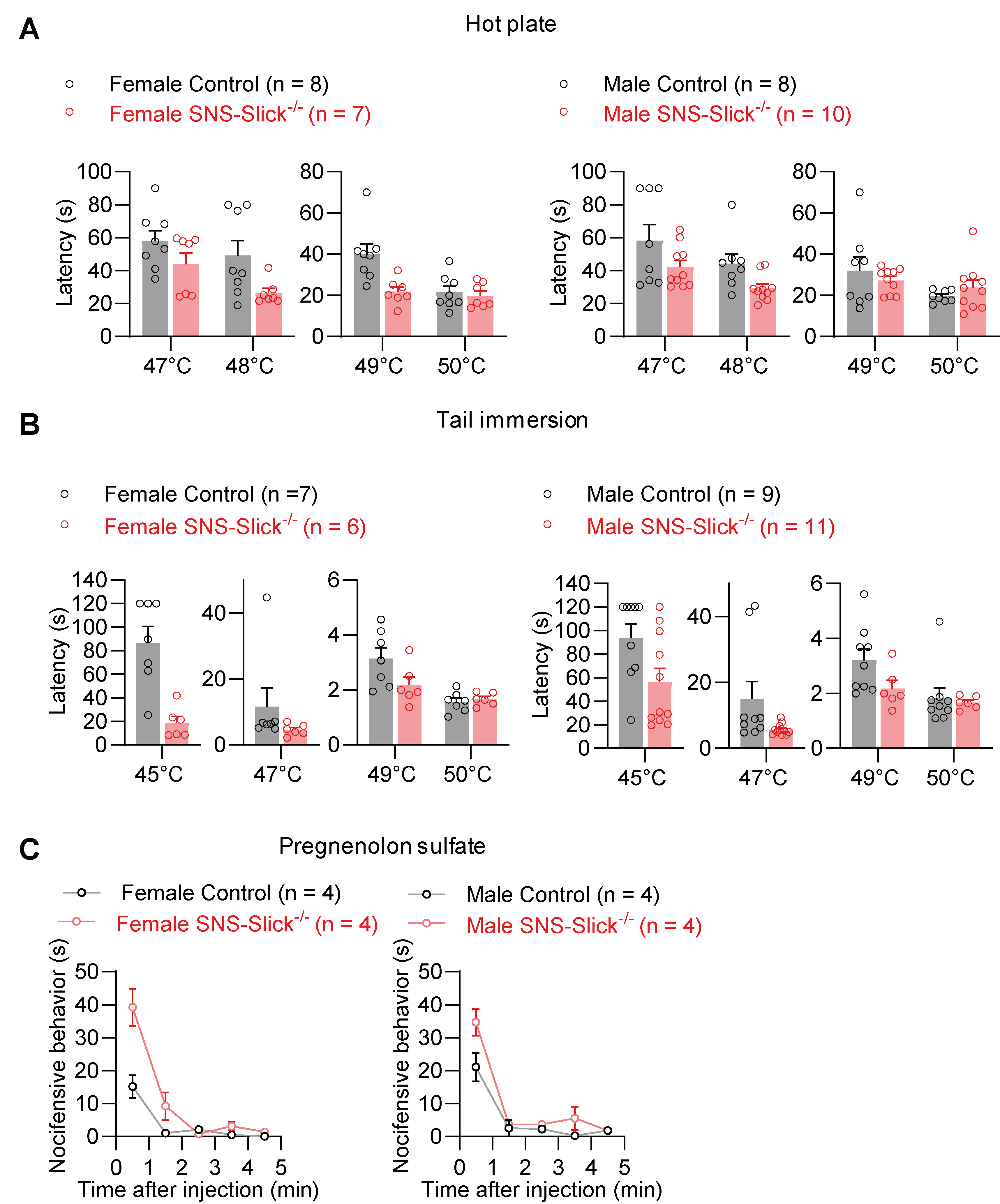

Supplement: Supplementary file 1 [file Image1.tif]
